# Barriers and facilitators to implementation of Universal Test and Treat in iLembe District, KwaZulu-Natal province: A qualitative study of healthcare workers’ perspectives

**DOI:** 10.4102/sajhivmed.v27i1.1825

**Published:** 2026-10-08

**Authors:** Mandla J. Zulu, Andrew Ross

**Affiliations:** 1 Department of Public Health, University of KwaZulu-Natal, Durban, South Africa; 2 Department of Family Medicine, University of KwaZulu-Natal, Durban, South Africa

**Keywords:** Universal Test and Treat policy, barriers and facilitators, effective implementation, stakeholder involvement, service delivery, health workforce, antiretroviral therapy, infrastructure and equipment

## Abstract

**Background:**

South Africa adopted the Universal Test and Treat (UTT) policy in 2016 to accelerate progress towards achieving the Joint United Nations Programme on HIV/AIDS (UNAIDS) 90–90–90 targets, which were later revised to UNAIDS 95–95–95 targets. However, implementation challenges have persisted with the iLembe District only achieving targets of 97–73–86 in 2024/2025.

**Objectives:**

The aim of this study was to explore the healthcare workers’ perspectives on the barriers and facilitators to the effective implementation of the UTT HIV policy in iLembe District in KwaZulu-Natal (KZN).

**Method:**

A qualitative, exploratory study was conducted across four clinics (rural, semi-urban and urban), with 12 professional nurses with ≥ 3 years’ experience in HIV care participating in face-to-face, semi-structured interviews. Audio-recorded interviews were transcribed verbatim and analysed deductively based on stakeholder involvement and WHO health systems building blocks.

**Results:**

Five predetermined categories – stakeholder involvement, service delivery, leadership, health workforce and medicines, infrastructure and equipment – were tested as barriers and facilitators to the UTT implementation in the iLembe District.

**Conclusion:**

Stakeholder involvement and adequate resources (human and other) to implement the UTT policy are an ongoing challenges to effective UTT implementation. Addressing these can help the iLembe District increase the number of new HIV tests, repeat tests and antiretroviral therapy initiations.

**What this study adds:** Multi-site qualitative insights from iLembe District identified HIV modifiable system bottlenecks. Practical actions tailored to district operations are proposed to optimise UTT towards achieving the 95–95–95 UNAIDS targets.

## Introduction

In 2016, South Africa (SA) adopted the WHO’s recommendation of using the Universal Test and Treat (UTT) policy to promote routine HIV testing with immediate initiation of antiretroviral therapy (ART) for all people living with HIV (PLHIV), irrespective of CD4 count. The purpose of this policy was to accelerate progress towards epidemic control by setting 90–90–90 targets for testing, treatment initiation and viral suppression.^1,2^ These targets were revised in 2020 to 95–95–95, with 95% of PLHIV knowing their status, 95% of those diagnosed with HIV receiving ART, of whom 95% achieve viral suppression.^3^ The UTT is a core strategy for achieving the Joint United Nations Programme on HIV/AIDS (UNAIDS) 95–95–95 targets.^3^ Evidence from randomised trials and programmatic evaluations indicates that earlier and broader ART use can reduce HIV incidence, improve survival and increase population-level viral suppression.^4^

Despite the UTT policy commitment, gaps persist along the HIV care cascade in SA and in KwaZulu-Natal (KZN) province, which has the largest number of PLHIV.^5^ A study conducted in the UThukela District municipality identified lack of emotional readiness, perceived medication side effects, stigma and poor socio-economic factors as barriers to UTT uptake and the fear of disease progression, acceptance of HIV status, support from family, positive testing experience and accessibility to ART treatment were identified as enablers to the implementation of the UTT policy.^6^

While the iLembe District in KZN implemented UTT in 2016, the district only achieved targets of 97–73–86 in 2024/2025 and barriers and facilitators of the policy have not been systematically explored.^7^ Achieving and sustaining the 95–95–95 targets requires reliable, context-specific implementation of UTT at the primary healthcare (PHC) level, where constraints in community engagement, workforce and infrastructure can undermine outcomes.^1,3^

Understanding the perspectives of the healthcare workers on the issues affecting the effective implementation of the UTT policy can illuminate operational bottlenecks, contextual barriers and enablers. The aim of this qualitative study was to explore healthcare workers’ perspectives on barriers and facilitators to effective UTT implementation in iLembe District across rural, semi-urban and urban settings to inform district-level improvements and contribute to the broader implementation literature.

## Research methods and design

### Study design and paradigm

This was a qualitative, exploratory study within an interpretivist paradigm, to understand the perceptions and experiences of healthcare workers in the iLembe District, and the contextual nuances of the UTT policy implementation.

### Setting

The study was conducted in four PHC clinics across iLembe District, KZN, that were selected to represent rural (Mbhekaphansi Clinic and Otimati Clinic), semi-urban (Mandeni Clinic) and urban (Ballito Clinic) contexts. Clinics provide comprehensive PHC services, including HIV testing, ART initiation, outreach and/or mobile services and community-based health worker (CHW) linkage.

### Sampling and participants

Using purposive sampling, 12 professional nurses (all women) were chosen who were directly involved in routine HIV testing, ART initiation and continuity of care. The nurses were chosen based on meeting the inclusion criteria, their availability and willingness to participate in the study. The sampling of the professional nurses took place at all four clinics (three participants per clinic), ensuring variation in geographic and service contexts. Consistent with the literature, a sample size of 12 was considered sufficient to explore the topic and to achieve data saturation.^7^

Inclusion criteria were professional nurses working in HIV, tuberculosis and sexually transmitted infection services in iLembe PHC clinics for ≥ 3 years and willing to participate in the study.

### Data collection

In person, semi-structured interviews were conducted in English by the researcher (first author) in March 2023, in a quiet clinic office at times that did not disrupt service delivery. The interviews followed a guide that explored stakeholder involvement, and four of the six WHO building blocks of a health system (service delivery, leadership; health workforce, medicines, infrastructure and equipment) and how these contribute (or not) to the implementation of the UTT policy. Stakeholders were considered to be the professional nurses providing the service and the clients that they serve.

### Data analysis

The audio-recorded data for each interview were transcribed verbatim to create transcripts for qualitative data analysis, to identify patterns of meaning across the data set and address the study objectives.^8^ A deductive approach was used, where the stakeholder involvement and the four WHO building blocks of a health system framework formed the basis of the analysis. The six phases of thematic analysis described by Clarke and Braun, which are widely used by many researchers in qualitative research, were used to analyse the data.^9^

As part of the analysis process, the researcher was reflexive, self-consciously critiquing and appraised the information presented.^10^ Being reflexive helped to bring into awareness the researcher’s opinions, feelings and experiences on the topic investigated, which helped to prevent bias. The researcher is a medical doctor and works in a District Hospital in the iLembe District, with many years of experience in the clinical management of HIV/AIDS and tuberculosis. This exposure could influence his conception and interpretation of the themes as participants express them, the act of being reflexive helped to prevent this from happening.

### Trustworthiness

Trustworthiness was ensured by creating an environment in which participants felt comfortable to participate, triangulation of the data (12 healthcare professionals from multiple sites), providing a detailed methodology, thick descriptions of how decisions were made, being reflexive, and by quoting directly from participants.

### Ethical considerations

Ethical clearance to conduct this study was obtained from the University of KwaZulu-Natal Biomedical Research Ethics Committee (No: BREC/00007668/2024) and the KwaZulu-Natal Department of Health (DoH) prior to data collection. Participants provided written informed consent and were assigned codes to protect their identity.

## Results

A deductive approach to data analysis was used based on the themes on the stakeholder involvement and the four WHO building blocks of a health system framework that describe the barriers and facilitators to effective UTT implementation: (1) stakeholder involvement; (2) service delivery; (3) leadership; (4) workforce; (5) medicines, infrastructure and equipment. The data supporting each theme are described below and depicted in Table 1. The participants were allocated a participant number and are reported as such, for example ‘P001, F, PN’ (Participant 1, Female, Professional Nurse).

**TABLE 1:** Themes and subthemes.

Themes	Sub-themes
1. Stakeholder involvement	Participants’ understanding of the UTT policy. Socio-economic impact and stigma on the UTT policy.
2. Service delivery	Immediate test and treatment of HIV. Clients’ attitudes towards repeated HIV testing. Clients’ attitudes towards the UTT policy. Age- and gender-specific patterns for HIV test and treat.
3. Leadership	A clear mandate. Provision of staff to implement the UTT policy (covered under Theme 4). Capacitation of staff (covered under Theme 4). Protocols for UTT.
4. Health workforce	Availability of staff to implement the UTT policy. Health worker training on the UTT policy.
5. Medicines, infrastructure and equipment	Availability of medicines for adults and children. Availability of infrastructure and equipment.

UTT, Universal Test and Treat.

### Theme 1: Stakeholder involvement

The stakeholders in this study were the 12 professional nurses who worked at the clinics and the clients that they serve. Their sound knowledge and understanding of the UTT policy ensured the implementation of UTT. However, they noted a number of barriers that were experienced by other stakeholders, their clients. These included stigmata related to testing and the impact of their socio-economic status, which prevented some of them from being able to afford food as well as the cost of transport to get to the fixed clinics for follow-up and to collect their medication. There were two subthemes under stakeholder involvement which were: (1) participant understanding of the UTT policy; and (2) socio-economic impact of the UTT policy.

All participants, as implementers, are important stakeholders of the UTT policy in the iLembe District and were knowledgeable about the UTT policy. They were able to articulate what it means to test and treat:

‘[*I*]f a patient tested positive then they have to start with treatment. You test and do pre and post counselling. Side effects caused by treatment must be explained to the patient and you give continuous counselling to the patient.’ (P001, F, PN)

When asked about the impact of socio-economic conditions on the willingness of clients to test for HIV, the participants reported that their clients often could not afford to pay for transport to return to the clinics to take repeat HIV tests:

‘Some are not working and they can’t even ask for help from their family, such as the transport fee to come to the clinic, because they don’t want to disclose their status.’ (P002, F, PN)

Some clients:

‘Don’t [*always*] have money to come to the clinic and collect treatment. They don’t have food to eat so they can take the treatment some are even resistant to take treatment because of economic challenges.’ (P003, F, PN)

Participants recognised that some clients don’t have food with which to take ART, and providing a relief or sick grant could assist PLHIV to buy food. Participants recommended that:

‘[*T*]he employer should give a grant to PLHIV because there are cases where you find that a client doesn’t even have food to eat so they can take treatment, even if it can be for a year at least some become stable after a year.’ (P001, F, PN)

### Theme 2: Service delivery

Service delivery issues related to ongoing testing and treatment, the former being necessary for diagnostic purposes to enable ART initiation to start as soon as a client tested positive. Four subthemes were identified under this theme: (1) immediate test and treatment of HIV; (2) clients’ attitudes towards repeated HIV testing; (3) client’s attitudes towards the UTT policy; and (4) age and gender patterns for HIV test-and-treat.

In general, participants felt that UTT implementation was facilitated by client’s knowledge regarding ART initiation, and their client’s positive attitudes toward HIV testing as well as repeated HIV testing. In terms of gender and age patterns, participants reported that more female than male clients test for HIV and that there was some reluctance among young people. Despite a general willingness to test, there were mixed opinions about the attitudes of different age groups and children infected by vertical transmission showing anger and resentment to their HIV status.

When asked about how soon patients should commence ART, the participants knew that, according to the UTT policy, they should start ART ‘[*a*]s soon as they get the results’, (P005, F, PN) and that if this was not possible, then ‘within 24 hours, because they don’t bring treatment during outreach, a client must be referred to the clinic. Sometimes they don’t come back to the clinic, which cause[s] delays’ (P006, F, PN).

Immediate ART treatment is offered to the clients who test positive for HIV at the clinics. However, delays occur when the HIV diagnosis is made by the outreach teams who do not keep medication to initiate ART in which cases clients are advised to come to the clinic to initiate ART.

When exploring their clients’ attitudes towards repeated HIV testing, the participants reported that ‘[*w*]e have never experienced any problem, since clients come willingly, some come expecting that they might be positive so they are ready to start treatment’ (P004, F, PN), and that ‘[*a*]fter counselling, they will have positive feedback; they agree to start treatment’ (P006, F, PN).

The participants felt that ‘most [*clients*] understand. Health education has helped, as they are taught the importance of knowing their status. Some refuse and deal with what they came for to the clinic’ (P002, F, PN).

In general, the participants agreed that:

‘90% of them don’t have a problem, 10% do have a problem. A person might come back sick to the clinic when HIV test is offered, they will say they did it last time.’ (P007, F, PN)

However, there are some:

‘Who have excuses, they will say they took a test on their last visit to the clinic, and they haven’t been sexually active. Even if you teach them about the window period they won’t agree and you can’t force them, they have to consent.’ (P001, F, PN)

There are times when clients:

‘[*G*]et angry, they don’t understand the ‘window period’ until it is explained to them properly, then they accept. It’s usually not met with a good attitude, because they don’t want to accept that they are HIV positive and need to take ART.’ (P008, F, PN)

Others ‘[*d*]on’t want to [*test*], even it is encouraged that every after three months they must be tested, but they always have excuses’ (P011, F, PN).

When clients do test positive, most:

‘[*U*]nderstand that they need to start treatment, there has been a few that were not taking it well as they never get a chance to process everything. It is all about health education, but most of them are accepting.’ (P009, F, PN)

Those who do test positive are ‘*m*ostly willing to start immediately’ (P010, F, PN).

However, a few ‘*d*on’t want to start immediately, they will say they need more time, although they are told that the longer you delay you are putting yourself at risk’ (P012, F, PN). Some clients ‘will say they are not yet ready, they need to think about it and they won’t come back’ (P001, F, PN), while others ’would cry to a point where you realise that if you give them treatment they won’t take it because they are still shocked’ (P002, F, PN).

Generally, clients were willing to test for HIV at facilities, while a few were hesitant to test for HIV but were persuaded with counselling and usually agreed to take the HIV test. ‘It depends which day you caught a client, most of the time they don’t have a problem’ (P012, F, PN). Although the feeling was that most of them like to test.

However, there was a concern about ‘people who are afraid to test’ (P009, F, PN), as those who ‘[*r*]efuse to test when they are asked, say they will come when they are ready’ (P002, F, PN) and that some of them ‘wait until a person is sick then they agree to test for HIV’ (P011, F, PN).

In terms of age and gender patterns, participants reported that more female than male clients test for HIV and that there was ambivalence among young people, with some willing and some reluctant to test. When women presented for family planning or antenatal care, they were generally willing to test for HIV, with a high percentage of pregnant women testing.

Despite a general willingness to test, there were mixed opinions about the attitudes of different age groups. Participants reported that:

‘[*Y*]oung people don’t easily accept, unless they had it in the back of their minds that they might be positive or if there is someone living with HIV in their home, it depends on the background.’ (P001, F, PN)

Others felt that ‘young people don’t want to be tested, others say they better off not knowing their status’ (P002, F, PN). Children infected by vertical transmission:

‘[*D*]on’t understand, seemingly they haven’t been taught well, especially those who are born with HIV, it is hard for relatives to explain the situation to them, so it is difficult for them to take treatment because they don’t understand what it is for. They also have anger issues because of how they got infected.’ (P004, F, PN)

However, other participants reported that ‘[*t*]he majority of the clients want to be tested, especially those who are around 18–24 years of age, but not necessarily all clients’ (P008, F, PN), ‘teenagers and preteens don’t have much problem with regards to testing’ (P012, F, PN), and that:

‘[*T*]he older ones are very negative towards testing because they know they participated in reckless behaviour. Female adolescents do have a problem, because they engage in reckless behaviour by sleeping with older men, so at the back of their mind they think they could be positive.’ (P001, F, PN)

There was, however, concern that men were often unwilling to test for HIV, and even if they tested:

‘[*M*]ales don’t want to take treatment. A female partner will test and find that they are positive, but when you ask them to bring their partner they will say he does not want to. If it happens that they come, then the female partner must always fetch treatment for them. Males don’t want to come to the clinic, even when they are fast tracked so they could come to the clinic they don’t want to.’ (P001, F, PN)

### Theme 3: Leadership

Leadership relates to issues of clarity of mandate, adequate staffing, capacitation of workforce; ongoing training and provision of protocols. Four subthemes were identified under this theme namely: (1) a clear mandate, (2) provision of staff to implement the UTT policy, (3) capacitation of staff and (4) protocols for UTT. Issues relating to staff will be covered under Theme 4.

The clinic leadership provided a clear mandate on the UTT policy and implementation so that all staff understood that ‘[*t*]hey are implementers for most of the programmes and information is being escalated to make everyone aware. There is also a policy in the clinic and training is provided’ (P007, F, PN).

In addition, the clinic management sets targets for the staff and monitors delivery as staff needed ‘[*t*]o test clients each and every day to reach the target set, if a person is positive they must be initiated immediately – the employer expects targets to be met’ (P005, F, PN).

The management is also responsible for conducting induction programmes to capacitate the staff for effective counselling and initiation of treatment. Participants felt that they had ‘[*s*]kills gained through inductions, such as learning how to do counselling, how do you assess the patients’ readiness’ (P003, F, PN). In addition, the clinic management provided guidelines and protocols for UTT and provides registers for data collection:

‘[*T*]hey make it a point that there are guideline policies in place and there is training to ensure that the policy is being implemented, registers are available and all the help they could offer.’ (P007, F, PN)

### Theme 4: Health workforce

Participants highlighted that there were shortages of nurses of all categories, including Nurse Initiated and Managed Antiretroviral Treatment (NIMART) trained nurses, with participants expressing the need for ‘[*m*]ore counsellors that deal with HIV’ (P007, F, PN) who would assist with one-on-one counselling to the clients. Two subthemes were identified under this theme, namely: (1) availability of staff; and (2) training of staff.

No additional staff were provided for the implementation of the UTT policy, with participants expressing the need for ‘[*m*]ore counsellors [*particularly*] in the high-volume clinics [*are needed*], because sometimes there is no[*t*] enough time to provide one-on-one counselling’ (P008, F, PN). They contended that to effectively run the UTT programme, there was a need to, ‘employ more staff, so they can do outreaches and visit communities so people can be taught about HIV’ (P001, F, PN).

Participants had either been trained formally on the UTT policy through training organised by the DoH and training colleges, through in-service training provided at the facility, or informally, with one participant learning about the UTT policy from social media. They indicated that ‘[*b*]efore we started with this work, they retrained or mobilised us to do UTT’ (P010, F, PN). Some participants wanted the DoH to send all health workers for training instead of only training a few professional nurses and expecting them to cascade the information by training others in the facility as ‘training must be available to every professional nurse, instead of sending one to represent a clinic and come back with feedback’ (P003, F, PN).

### Theme 5: Medicines, infrastructure and equipment

Two subthemes were identified under this theme, namely: (1) availability of medicine; and (2) availability of infrastructure, equipment and transport.

In terms of availability of medications for adults and children, adult ART treatment is generally available, while for children it is out of stock from time to time, with participants stating that:

‘For now it is available, though there is a shortage of ART for children. Sometimes they are given monthly treatment, which becomes a challenge because some can’t afford to come to the clinic every month.’ (P001, F, PN)

Participants reported that there is a need for management to, ‘ensure that the treatment is always available for the clients’ (P003, F, PN). There was a feeling that, ‘there should be more stock, especial to treat children, because sometimes we run out of stock’ (P006, F, PN).

In terms of infrastructure and equipment, the infrastructure was largely regarded as being suitable for the participants to offer counselling, testing and treating and suitable equipment was available. Participants felt that they were able to offer counselling and testing and treating the clients except in a few cases, such as:

‘On a doctor’s day a doctor comes and occupies the consultation room so in the next room there will be two of us working in there. If a patient asks to be tested, there is no way to test because there is no privacy and this affects the clients’ rights to privacy and confidentiality.’ (P008, F, PN)

Although others reported that private spaces are, ‘available, unless they have certain programmes in those rooms, then we move to the vital station room’ (P003, F, PN).

Participants reported that HIV test strips were available, ‘since we won’t be able test without testing kits being available’ (P001, F, PN).

Management was also responsible to ensure, ‘material to take bloods is available’ (P003, F, PN) as well as registers used for record.

However, they expressed a need ‘for those viral unsuppressed clients they can be traced telephonically using a phone from work and then allocate more staff for tracing’ (P003, F, PN).

## Discussion

This multi-site qualitative study in iLembe District has highlighted some of the facilitators and barriers to implementing the UTT policy. Addressing these factors has the potential to improve the UTT policy implementation and subsequently achieve the UNAIDS 95–95–95 targets.

Professional nurses working in the clinics are key stakeholders in implementing the UTT policy and their buy-in is important to ensure the successful implementation of the policy. Their knowledge and experience are essential to facilitating the implementation of the testing and treating of HIV. This finding is consistent with that of Nicol et al. that was conducted in the adjacent UThukela District.^11^ The current study highlights the important role that the nurses play in ensuring that the implementation process runs smoothly in clinics and in community outreach, as they know what needs to be done at all stages to ensure opportunistic HIV testing and ART treatment is provided to clients. Within the DoH there is little opportunity for healthcare workers to provide feedback about the challenges that they face in the implementation of policy. This study has highlighted some of the factors that healthcare workers felt negatively influenced the implementation of the UTT policy. These included issues such as staff shortages, paediatrics ART unavailability, the need for more training, mobile units’ shortages of medicine, infrastructural challenges, as well as HIV-associated stigma in the community and the effects of poverty on the uptake of the UTT. It is hoped that research projects such as this can highlight these issues with the relevant authorities in the DoH.

Clients are another important stakeholder in the implementation of the UTT policy. Socio-economic realities and HIV stigma in the iLembe District emerged as barriers for HIV testing and treatment uptake, with some clients being reluctant to ask for transport fare from relatives and friends to test or take a retest at the clinics. The failure to retest is a setback in the diagnosis of new HIV infections, especially to those individuals who are in a window period during the initial test. In addition, participants reported that some clients indicated that they do not have food as a cause for not initiating ART, as the medication needs to be taken with food.^6^ A study in the adjacent King Cetshwayo District also identified the lack of transport money as well as the lack of food as barriers to effective implementation of the UTT policy.^11^

HIV stigma emerged as a barrier to the test and treat as clients feared being stigmatised once their status was revealed or when their reasons clinic visits were known to their families. This fear resulted in clients not returning to the clinic for repeat tests, which is in keeping with another study which reported high HIV stigma in SA and the impact that this has on testing and adherence.^12^ HIV stigma will remain a barrier to the UTT policy being successfully implemented if not addressed by decision-makers and non-government organisations in KZN.

It is encouraging, however, that the majority of clients attending the PHC facilities were willing to take HIV tests multiple times and to commence ART immediately upon HIV diagnosis. This is a facilitator to the effective implementation of the UTT policy and concurs with the DHIS finding of 97% of iLembe District.^13^ These performance data are higher than the UNAIDS target of 95% of PLHIV needed to be on ART to achieve the 2030 Sustainable Development Goals.^3^ However, it is of concern that despite the high number of clients testing, only 73% actually initiated treatment in 2024/2025 in iLembe,^13^ highlighting the need to address factors that deter clients from initiating treatment.

Participants reported that most women of all ages were willing to test for HIV repeatedly and to start ART treatment immediately if they tested positive. This is a facilitator to the effective implementation of the UTT policy and concurs with the findings of a study conducted in the adjoining eThekwini District Municipality, which showed that same-day initiation varied between 60% and 82%.^5^ Although encouraging, this is well below the UNAIDS target of 95% of PLHIV needed to be on ART to achieve the 2030 Sustainable Development Goals. Studies conducted in Senegal and Zimbabwe show similar results.^14^ With HIV prevalence of 9.1% in young women compared to 3.1% for young men it is essential for all women, especially those who are young and pregnant, to test for HIV and start ART as soon as possible to reduce vertical transmission.^15^ It was encouraging that participants reported the willingness of young people to test for HIV, which will assist in identifying new cases of PLHIV in this age group.

However, participants expressed concern about target groups, such as men, who are reluctant to test for HIV and generally do not want to go to the clinics, often waiting to seek medical help at an advanced stage of the disease when it is difficult to treat. This is consistent with the study by Sabo et al., which showed that men were significantly less likely to test than women where male sex had an adjusted odds ratio of 0.51 (95% confidence interval: 0.49–0.53).^16^ Participants cited inadequate outreach programmes, insufficient staff and transport resources as some of the barriers limiting initiatives to identify and test men, making it essential to find innovative ways to reach test and treat them early to prevent more infections and disease progression in those already infected with HIV.

The clinic leadership provided a clear mandate on the UTT policy which all staff understand. All healthcare workers were knowledgeable about the UTT policy which they acquired through induction, in-service training or through other health workers who attend training and cascaded the information to them. Other studies have also reported similar findings about health workers’ clear knowledge of the UTT.^3^

Participants reported that the clinic management sets clear HIV test and treat targets for the staff and monitors delivery. The target setting and regular discussions on HIV testing and treatment ensure that staff monitor and report on the number of clients who come the clinic who are tested for HIV, those who test positive as well as those who are initiated on ART. In addition, the clinic management provided tools for the successful implementation of UTT through the provision of guidelines and protocols, as well as registers for data collection. These findings are different from those of a study by Mnyaka et al., where the lack of leadership was shown to be a barrier to UTT implementation.^2^

It is important that management provides an enabling environment for the workers in accordance with the WHO technical brief findings that the attitudes and behaviours of health workers can be a barrier to UTT.^3^ Implementation of the UTT policy in iLembe District is provided by the same health workers offering other clinic services, which resulted in their being overstretched by the additional duties. The lack of sufficient personnel resulted at times in inadequate counselling for the clients who were tested, which led to some patients’ being reluctant to take the treatment and others defaulting. Other studies have also identified the shortage of staff as a barrier to the UTT policy implementation.^17^ The shortage of all categories of nurses, including those who are NIMART trained, was also reported in other parts of the country, such as the Eastern Cape province.^2^

Most participants reported that they had been trained on UTT, either directly or with the DoH selecting some healthcare workers to attend training workshops, and who then had to train the rest of the staff upon returning to the facilities. In the current study participants expressed confidence in their knowledge of UTT and their ability to test, re-test and initiate clients on ART. A study conducted in Rustenburg, North West province, also found that the staff had adequate knowledge of UTT and had HIV treatment guidelines available, which contrasts with the findings of Mnyaka et al., who reported that very few nurses were trained on the NIMART programme.^2,17^

The availability of adult ART was reported to be satisfactory most of the time, except when national shortages occurred, while stock-outs for paediatric ART were reported in most facilities from time to time. The medicines stock-outs affect the same-day treatment of patients who test positive for HIV and at times result in non-compliance with ART, as Filiatreau et al. highlighted; the challenges associated with stock-outs impacts on achievement of the 95–95–95 UNAIDS targets.^18^ The Provincial and National DoH need to make effective use of the stock-out dashboard information to monitor the availability of stock in all facilities and ensure that stock is always available.

The lack of adequate health facility workspace was a challenge for some facilities in the iLembe District, with nurses having to share consulting rooms on the days that the Medical Officers visit the clinics, resulting in difficulty keeping privacy and confidentiality during the counselling and testing sessions with patients.

Participants also reported challenges with reaching clients in communities outside the clinics because of insufficient vehicles and staff shortages, these being the main barriers to conducting community outreach services in order to test, retest and treat clients who were unable to come to the clinics for a variety of reasons. The availability of fully capacitated mobile units will enable the unreachable clients in both urban and rural settings in the iLembe District to be accessed.

### Recommendations

The iLembe District and the KwaZulu-Natal DoH should capacitate the clinics with sufficient and well-trained workforce to ensure successful implementation of the UTT policy. Overwork and burnout of staff will lead to staff turnover and affect the UTT policy implementation.^19^

The provision of more fully capacitated mobile units will improve the test and treat of the clients in the proximity of their homes, accessing both adults and children. The mobile units will also be able to access farm workers as well as general community members who cannot get to the clinics during working hours. The mobile units will also increase the opportunities for men to access services to enable timeous testing and ART initiation.

Standardised up-to-date medication supply information technology systems need to be implemented to ensure that stock-outs do not occur, as this can affect adherence to ART.

The use of CHWs should be optimised to extend the UTT beyond facilities to people’s homes. The CHW programme has proven effective for delivering community-based health services in SA since the programme was formalised in 2011.^20^ In addition, those who are stable on ART need to be able to get access to at least six month supply of tablets. This would prevent them having to spend a day at the clinic each month, which can incur transport costs that they cannot afford, result in losing pay for those who are employed and missing appointments because of other household responsibilities.

The DoH should consider conducting campaigns to educate communities about the importance of test and treat for HIV to destigmatise HIV. In addition, schools should be encouraged to allocate the role of HIV counsellors to teachers who children can speak to about their concerns related to their HIV status and can either accompany them to the clinic or give them information about where to go to ensure that they access the services they need.

The South African government should consider providing an HIV relief grant for PLHIV to allow them to buy food to take their treatment.

### Strengths and limitations

Strengths of this study were the inclusion of multi-site sampling across diverse geographies, selection of experienced nurses deeply embedded in HIV service delivery and triangulation across settings, thereby enhancing credibility. While the qualitative findings are context specific and not transferrable to other settings, they offer transferable lessons for similar districts.

## Conclusion

The healthcare providers’ perspectives and experiences of implementing UTT in the iLembe District are varied but show their good understanding of the policy and have highlighted some of the barriers and facilitators affecting its uptake among clients they serve. Ideally policymakers should encourage feedback from healthcare workers in clinics as they are the backbone of and important stakeholders in the implementation of the UTT policy. The provision of adequate counselling and medicines for children needs urgent attention. A community-wide strategy to educate clients and especially men about the UTT policy will reduce avoidable morbidity and mortality from HIV/AIDS in the iLembe District and improve the quality of life for all citizens.
